# Development and optimization of a methimazole microemulsion for topical application: Formulation characteristics and transdermal permeation

**DOI:** 10.1111/jocd.16528

**Published:** 2024-08-12

**Authors:** Anayatollah Salimi, Hadis Hoseinzadeh, Saeed Mohammad Soleymani

**Affiliations:** ^1^ Department of Pharmaceutics, Faculty of Pharmacy Ahvaz Jundishapur University of Medical Sciences Ahvaz Iran; ^2^ Nanotechnology Research Center Ahvaz Jundishapur University of Medical Sciences Ahvaz Iran; ^3^ Department of Clinical Pharmacy, School of Pharmacy Shahid Beheshti University of Medical Sciences Tehran Iran; ^4^ Clinical Research Development Centre, Imam Hossein Educational Hospital Shahid Beheshti University of Medical Sciences Tehran Iran

**Keywords:** melasma, methimazole, microemulsion, optimization, permeability

## Abstract

**Background:**

Methimazole, an oral antithyroid drug, has recently gained attention for its skin‐brightening effects when applied topically to treat melasma. This study aims to develop, optimize, and characterize a methimazole microemulsion as a novel, safe approach for local melasma treatment.

**Materials and Methods:**

We prepared microemulsion formulations containing 3% methimazole by combining appropriate amounts of surfactants (Tween 80 and Span 20), propylene glycol cosurfactant, and an oil phase (oleic acid‐transcutol p at a 1:10 ratio). We then assessed droplet size, stability, viscosity, and skin permeation using rat skin models.

**Results:**

The microemulsions' droplet sizes ranged from 7.06 to 28.13 nm, with viscosities between 120 and 254 centipoises. Our analysis identified droplet size, viscosity, and membrane release as significant independent variables. We determined the permeability parameters of the optimal formulation through rat skin, including steady‐state permeability rate (J_ss_), permeability coefficient (p), lag time (T_lag_), and apparent diffusion coefficient (D_app_).

**Conclusion:**

We found that the microemulsions' characteristics, physicochemical properties, and in vitro release depended on the surfactant‐to‐cosurfactant ratio, water content, and oil content. We developed an optimal formulation with a high surfactant‐to‐cosurfactant ratio and low water and oil percentages. This formulation shows potential for commercialization and manufacturing of final products.

## 1 INTRODUCTION

Melanogenesis is a complex physiological process resulting in the production of melanin, a pigmented biopolymer derived from tyrosine. Melanin is synthesized in melanosomes, organelles related to lysosomes in melanocytes, and protects the skin from the harmful effects of sunlight, toxic drugs, and chemicals.^1^, ^2^

Besides determining skin color and influencing appearance, abnormally high melanin production can lead to hyperpigmentation disorders. While usually harmless, increased pigmentation, especially on the face (such as melasma and freckles), can significantly impact a person's appearance. This may cause emotional and psychological distress, affecting quality of life. Melasma, a common acquired hypermelanosis, typically occurs in sun‐exposed areas, primarily on the face, occasionally on the neck, and rarely on the arms.^3^

Melasma treatments focus on protection from ultraviolet rays and reducing epidermal melanin. Hydroquinone (HQ) is the primary ingredient in topical agents for hyperpigmentation disorders. However, HQ often causes side effects and has cytotoxic and mutagenic effects.^4^

Methimazole (1‐methyl‐2‐mercaptoimidazole), with the molecular formula C4H6N2S, is a thioamide that inhibits the thyroid peroxidase enzyme. It has antithyroid activity and is used to treat hyperthyroidism.^5^ Side effects of methimazole include toxicity and liver damage.^6^

When applied topically, methimazole inhibits skin melanin production, acting as a depigmenting agent in both laboratory animals and humans. It interferes with various stages of eumelanin and pheomelanin synthesis by inhibiting peroxidase in skin melanocytes and blocking the metabolism of several melanin mediators, including dihydroxyphenylalanine, dihydroxyindole, and benzothiazine.^7^ Unlike hydroquinone, topical methimazole has no cytotoxic or mutagenic effects and does not affect plasma thyroid hormone levels.^8^

Applying drugs to the skin is a method of drug delivery that aims to achieve both local and systemic effects. This approach offers several advantages, such as avoiding first‐pass hepatic metabolism, providing continuous and controlled drug delivery, reducing dosing frequency, and increasing patient acceptance.^9^

Microemulsions (MEs) are stable colloidal systems with droplet sizes smaller than 100 nm. They consist of oil and water stabilized by a mixture of surfactants and cosurfactants.^10^ As liquid and isotropic formulations, MEs have been extensively studied as drug delivery systems for various routes, including skin application. Their advantages over conventional unstable emulsions include ease of preparation, thermodynamic stability, enhanced penetration, and transparency.^11^

This research aims to develop an ME pharmaceutical form of methimazole with appropriate therapeutic efficiency, designed to increase the drug's effectiveness and stability at the target site.

## 2 MATERIALS AND METHODS

### 2.1 Materials

The following materials were obtained for this study: methimazole (Iran Hormon Company); propylene glycol, Span 20, Tween 80, oleic acid, sodium dihydrogen phosphate, and sodium hydrogen phosphate (Merck, Germany); Transcutol P (Gattefosse, Germany); and cellulose membrane (Armaghan Kala Jounob Company, Iran).

### 2.2 Animals

This study was approved by the Ethics Committee of Jundishapur University of Medical Sciences (IR.AJUMS.ABHC.REC.1397.087) on March 13, 2019. Adult male Wistar rats (150–170 g, 10–12 weeks old) were used. The rats were anesthetized and euthanized using ketamine (80 mg/kg) and xylazine (10 mg/kg). After confirming death, abdominal hair was removed using an electric shaver. The abdominal skin was carefully excised using scissors and tweezers. Healthy‐looking skin samples were cleaned of fat using cold acetone‐soaked cotton. Skin thickness was measured with a digital micrometer. The samples were then wrapped in aluminum foil, labeled with diameter and preparation date, and stored below −20°C.^12^

### 2.3 Methimazole measurement

A spectrophotometer was used to measure methimazole at 251 nm. This wavelength was selected based on methimazole's absorption spectrum in phosphate buffer (pH 7.4), where it showed maximum absorption without interference from other substances.^13^, ^14^

### 2.4 Phase diagram

Pseudo‐ternary phase diagrams were created based on previous studies. The components included:

- Surfactants: Span 20 (HLB 8.6) and Tween 80 (HLB 15).
- Cosurfactant: Propylene glycol.
- Oil phase: Oleic acid and Transcutol P (ratios 3:1 and 2:1).

Using factorial design and pre‐formulation trials, eight formulations were selected with three variables at two levels each (Table 1). The variables were:

- Surfactant to cosurfactant ratio (3:1 and 1:2).
- Oil proportion (5% and 50%).
- Water content (5% and 10%).

**TABLE 1:** Methimazole ME formulation components.

Formulation	Factorial	S/C	% Oil	% S + C	% methimazole	Water
ME‐MTZ‐1	+ + +	3:1	50	37	3	10
ME‐MTZ‐2	− + +	3:1	50	42	3	5
ME‐MTZ‐3	+ − +	3:1	5	82	3	10
ME‐MTZ‐4	− − +	3:1	5	87	3	5
ME‐MTZ‐5	+ − −	2:1	5	82	3	10
ME‐MTZ‐6	− − −	2:1	5	87	3	5
ME‐MTZ‐7	− + −	2:1	50	42	3	5
ME‐MTZ‐8	+ + −	2:1	50	37	3	10

**FIGURE 1:**
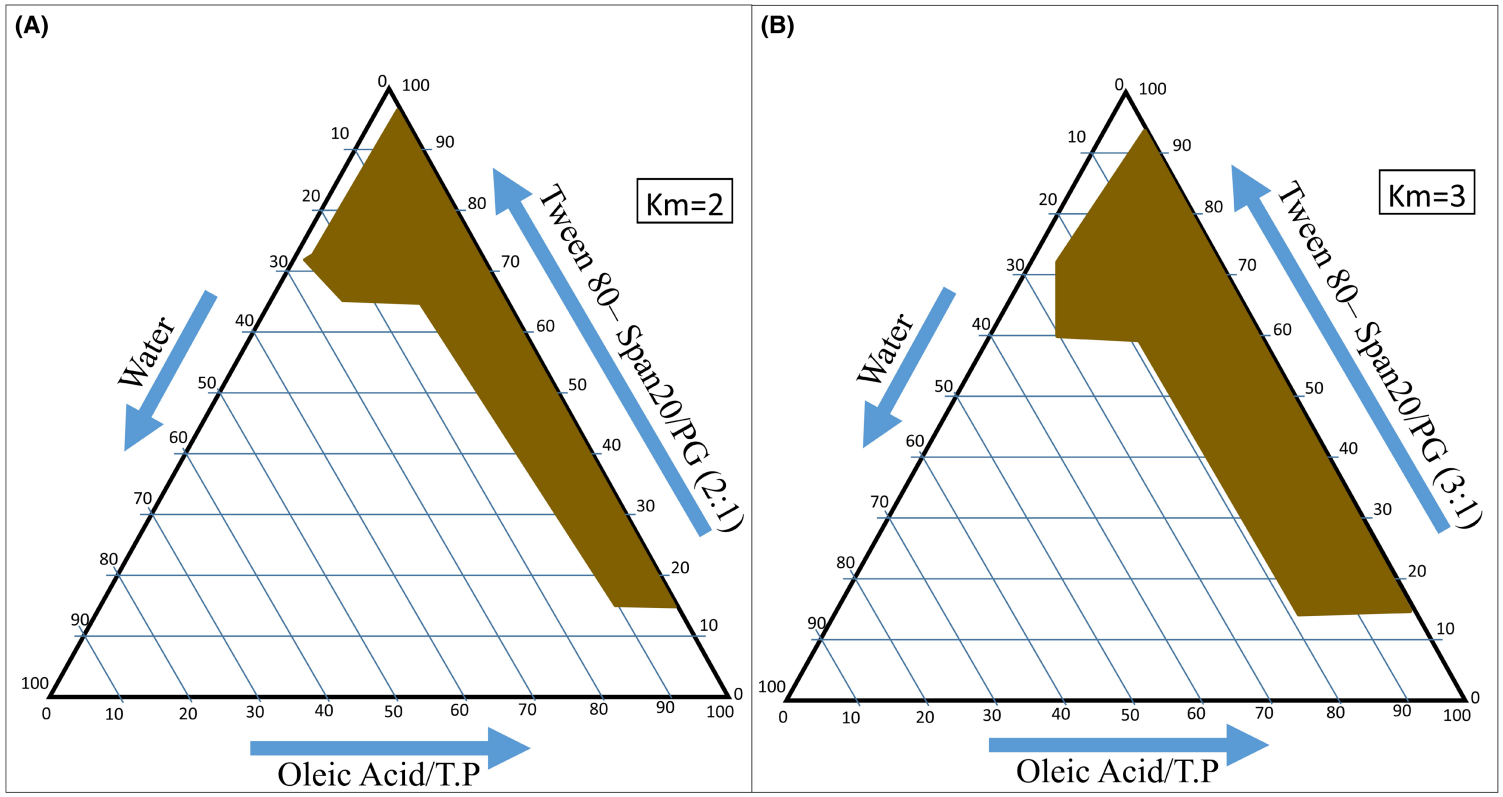
Ternary phase diagrams for non‐ionic surfactant to cosurfactant ratios of (A) 2:1 and (B) 3:1.

Each formulation contained 3% of the drug. Figure 1 shows the phase diagram used in this study.^15^

Using a factorial design and pre‐formulation tests, we selected eight formulations based on three variables, each at two levels (Table 1). The variables in this study were:

- Surfactant to cosurfactant ratio (3:1 and 2:1).
- Oil percentage (5% and 50%).
- Water content (5% and 10%).

We added 3% of the drug to each formulation and conducted analyses.^15^

### 2.5 MEs preparation

We prepared methimazole‐containing MEs by first adding the drug to the oil mixture, and then combining this with the surfactant and cosurfactant mixture. We then added distilled water dropwise while stirring to obtain the final ME.^16^

### 2.6 Evaluation of ME droplet size

We analyzed the droplet size of each ME using a Particle Size Analyzer. The average droplet size and dispersion index were measured by Laser Light Scattering at 25°C.^17^

### 2.7 Evaluation of viscosity and pH of drug‐containing samples

We measured the viscosity of selected samples at 25°C using a Brookfield viscometer model DV‐II with spindle 34. Measurements were taken in 10 mL volumes at shear speeds of 50, 75, and 100 rpm.^18^

We measured pH using a Mettler pH meter at 25°C without dilution.^19^

### 2.8 Evaluation of ME stability

We prepared 5 mL volumes of several formulations and stored them at 4°C, 25°C, and 37°C for 6 months. We visually examined the samples weekly for phase separation, transparency, and sediment formation. Any changes, such as turbidity or phase separation, indicated instability.^20^

### 2.9 Evaluation of drug release

We used a standing Franz diffusion chamber (cross‐sectional area: 4.906 cm^2^) to assess drug release from different formulations. We used phosphate buffer (pH 7.4) as the receiving phase and a cellulose synthetic membrane (soaked in deionized water for 24 h before use) as the membrane model.

We filled the receiver chamber with 35 mL of receiving phase and placed it on a stirrer at 37 ± 0.5°C, with a magnet at 200 rpm. We spread 5 g of each formulation on the membrane. At regular intervals (0.5, 1, 2, … 8, and 24 h), we removed 2 mL from the receiver chamber and replaced it with a fresh solution. We determined the amount of released drug using spectrophotometry at 251 nm.^12^

### 2.10 Evaluation of methimazole permeation through rat skin

**FIGURE 2:**
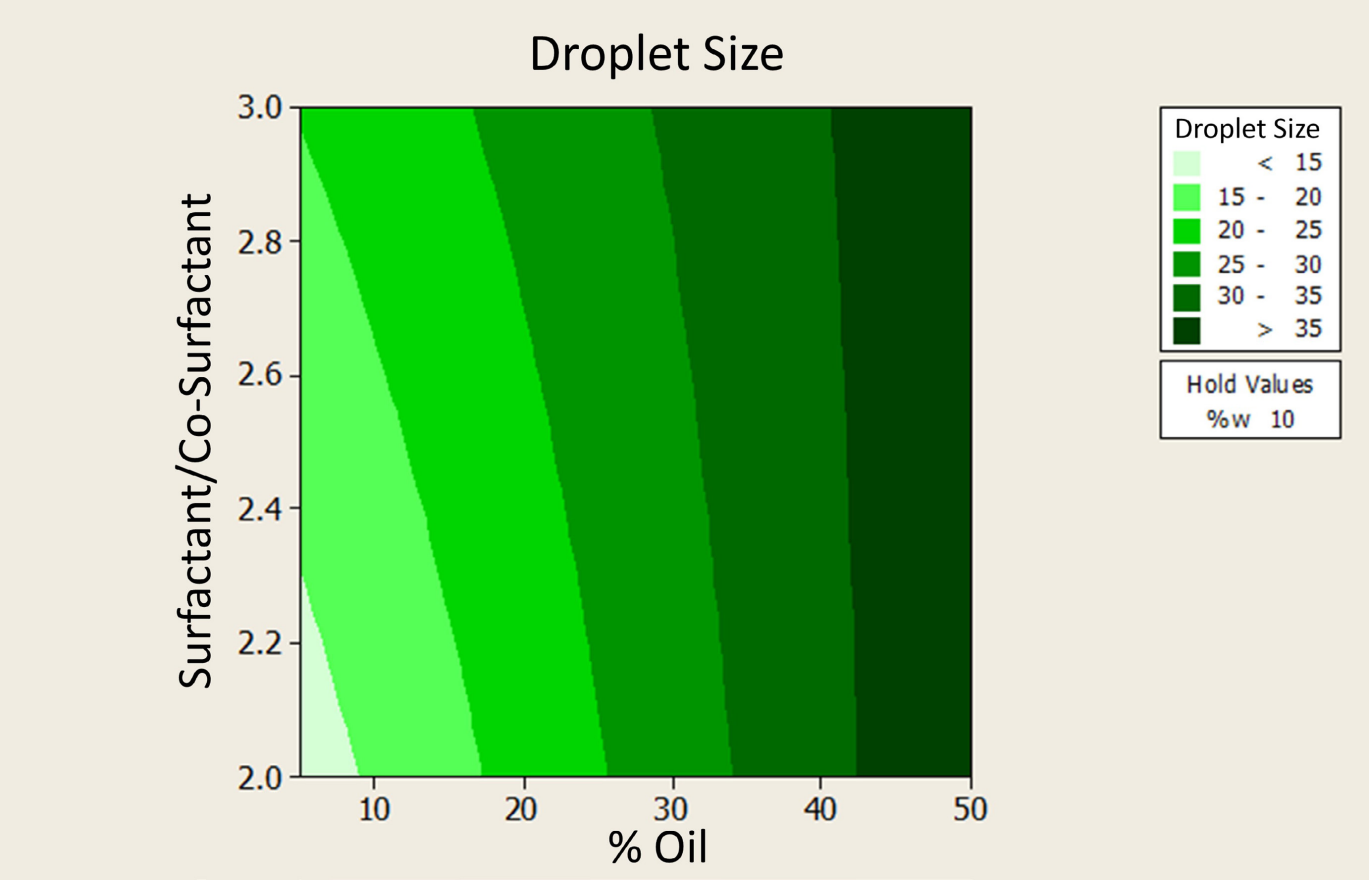
ME droplet size vs. oil percentage and surfactant/co‐surfactant ratio.

To assess skin permeability, we placed 5 g of ME formulation on hydrated rat skin in the donor phase of Franz cells. We filled the receiver phase with phosphate buffer (pH 7.4) and stirred it at 200 rpm. We sampled the receiver phase at designated times (0.5, 1, 2, … 8, and 24 h), removing 2 mL and replacing it with fresh buffer to maintain sink conditions. We measured drug permeation spectroscopically at 251 nm, using a 3% aqueous suspension of methimazole as a control (Figure 2).^18^

We investigated methimazole permeation from MEs through whole rat skin and calculated permeation parameters including steady‐state permeability rate (Jss), permeability coefficient (p), lag time (Tlag), and apparent diffusion coefficient (Dapp) (Table 2). We also calculated ERflux, ERD, and ERp of drug‐containing MEs compared to the drug‐saturated control (Table 2).

**TABLE 2:** Physicochemical properties of methimazole‐containing MEs (*n* = 3, Mean ± SD).

Formulation	Droplet Size (nm)	Polydispersity Index (PDI)	Droplet Size (nm) after 6 months	Viscosity in 50 rpm (cps)	Viscosity in 75 rpm (cps)	Viscosity in 100 rpm (cps)	pH	pH after 6 months
ME‐MTZ‐1	38.8 ± 1.2	0.39 ± 0.02	39.0 ± 0.5	140 ± 1	137 ± 1	134 ± 1	4.32 ± 0.01	4.40 ± 0.02
ME‐MTZ‐2	39.5 ± 2.5	0.38 ± 0.01	39.8 ± 0.2	145 ± 2	148 ± 4	154 ± 4	4.38 ± 0.01	4.45 ± 0.05
ME‐MTZ‐3	47.0 ± 2.2	0.39 ± 0.01	47.3 ± 0.1	229 ± 2	227 ± 1	224 ± 2	4.66 ± 0.02	4.60 ± 0.08
ME‐MTZ‐4	37.6 ± 2.4	0.40 ± 0.02	38.0 ± 0.0	254 ± 3	237 ± 3	241 ± 3	4.61 ± 0.01	4.65 ± 0.01
ME‐MTZ‐5	41.7±/1.3	0.41 ± 0.01	41.9 ± 0.5	142 ± 1	139 ± 2	133 ± 1	4.42 ± 0.03	4.50 ± 0.01
ME‐MTZ‐6	9.3 ± 0.3	0.41 ± 0.01	9.4 ± 0.2	201 ± 2	198 ± 3	194 ± 4	48.4 ± 0.01	4.50 ± 0.01
ME‐MTZ‐7	12.7 ± 1.1	0.40 ± 0.01	12.8 ± 0.1	130 ± 1	126 ± 1	124 ± 2	4.49 ± 0.02	4.52 ± 0.02
ME‐MTZ‐8	20.2 ± 0.9	0.39 ± 0.01	20.5 ± 0.3	120 ± 3	115 ± 2	114 ± 1	4.43 ± 0.02	4.51 ± 0.03

*Note*: Methimazole ME number 3 has the largest droplet size, while ME number 6 has the smallest. We found a significant relationship between droplet size and both the surfactant‐to‐cosurfactant ratio and oil percentage. As these ratios increase, so does the droplet size.

To calculate permeability parameters, we plotted the cumulative amount of drug permeated per unit area against time. We obtained Jss by multiplying the permeability coefficient (p) by the drug concentration in the donor phase (C). We calculated the apparent diffusion coefficient (Dapp) by dividing the square of the skin diameter (h) by 6 times the lag time (T_lag_).^15^, ^21^

Since h doesn't represent the actual drug passage length, D calculated from this formula is apparent. As all calculations were based on the steady‐state region of the cumulative drug permeability diagram, sink conditions were necessary for reliable parameters. In this study, the maximum concentration in the receptor phase was less than 10% of the drug's saturated solubility, ensuring a constant concentration gradient and passage rate during experiments.^15^

### 2.11 Statistical analysis

We repeated all experiments three times and expressed values as means with standard deviations. We used two‐sided *t*‐tests and analysis of variance for statistical analysis, with significance set at *p* < 0.05. We designed the Full‐Factorial test using Minitab 16 software. We calculated the optimal formulation using the optimization method in Minitab 16.^15^

## 3 RESULTS

### 3.1 Physicochemical properties of methimazole MEs

Table 2 shows the physicochemical characteristics of methimazole‐containing MEs. These include:

1. Droplet size on the day of manufacture and after 6 months (stability).
2. Polydispersity index.
3. Viscosity at three different shear rates.
4. pH on the day of manufacture and after 6 months (stability).

MEs 8 and 4 show the lowest (115 cps) and highest (237 cps) viscosity, respectively. The formulations' viscosity correlates significantly with oil percentage, water percentage, and surfactant‐to‐cosurfactant ratio. Viscosity increases when:

1. Oil percentage rises.
2. Water percentage decreases.
3. Surfactant to surfactant‐to‐cosurfactant ratio decreases.

The pH of MEs has a strong link to water percentage. As water content increases, pH decreases. Our results indicate that ME 3 has the highest pH, while ME 1 has the lowest.

### 3.2 Drug Release

**FIGURE 3:**
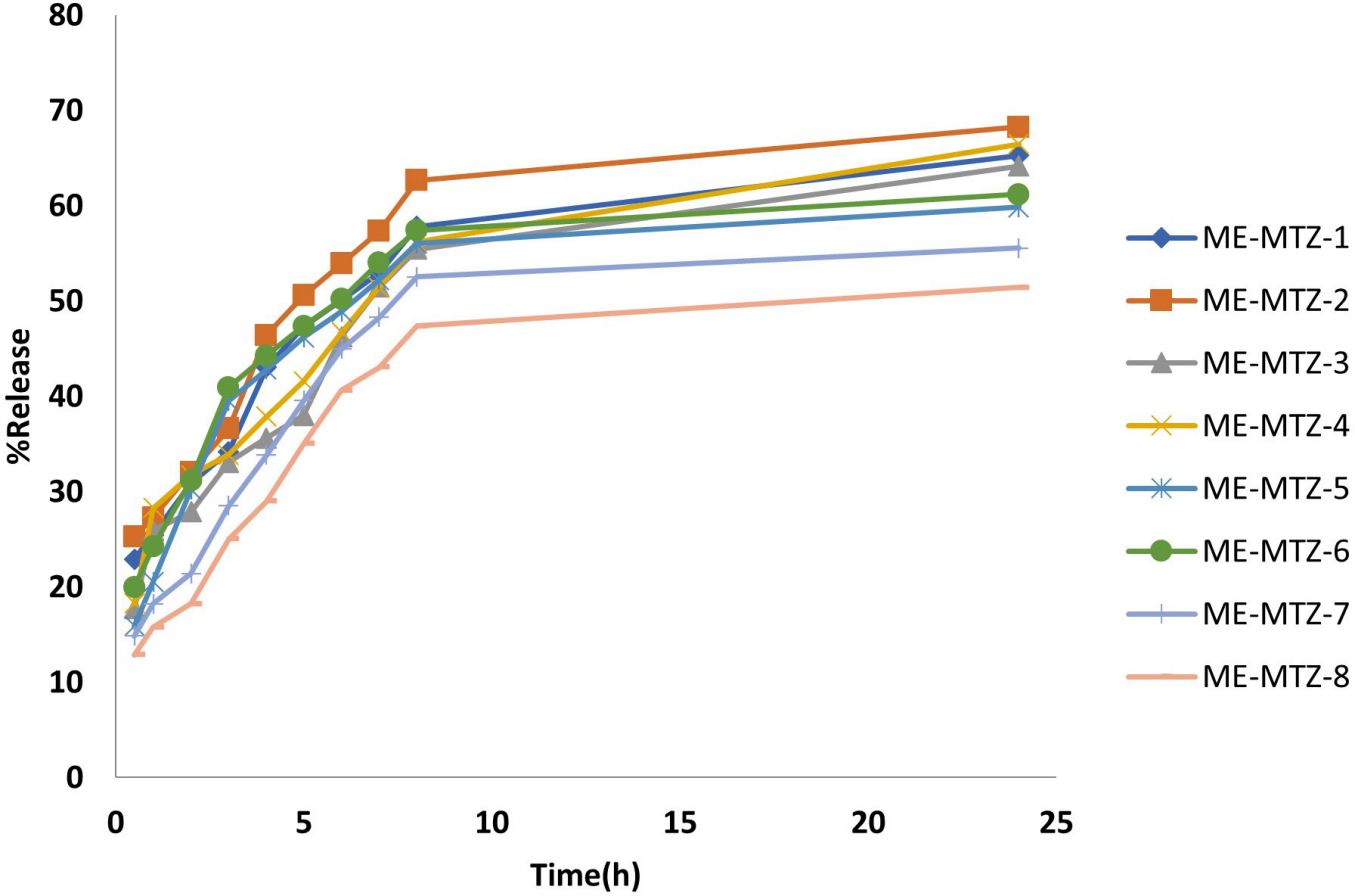
Methimazole cumulative release rate from ME formulations.

Table 3 and Figure 3 present the rate and mechanism of drug release from MEs. Our findings show that over 24 h, formulation number 2 released the highest percentage of the drug, while formulation number 8 released the lowest.

**TABLE 3:** Drug release mechanism results from ME formulations (*n* = 3, Mean ± SD).

Formulation	Kinetic model	*r* ^2^	Q_2h_ ^a^_(%)_	Q_24h_ ^b^_(%)_
ME‐MTZ‐1	Higuchi	0.8688	30.89 ± 0.05	65.29 ± 0.09
ME‐MTZ‐2	Higuchi	0.8385	23.07 ± 0.06	68.27 ± 0.56
ME‐MTZ‐3	Higuchi	0.8883	27.89 ± 0.06	64.16 ± 0.21
ME‐MTZ‐4	Higuchi	0.9103	31.75 ± 0.06	66.46 ± 0.04
ME‐MTZ‐5	Higuchi	0.7793	3021 ± 0.04	59.85 ± 0.04
ME‐MTZ‐6	Higuchi	0.7921	31.18 ± 0.12	61.17 ± 0.02
ME‐MTZ‐7	Higuchi	0.8022	21.36 ± 0.04	55.53 ± 0.38
ME‐MTZ‐8	Higuchi	0.8227	18.25 ± 0.04	51.42 ± 0.06

^a^ Q_2h_: 2‐h drug release.

^b^ Q_24h_: 24‐h drug release.

Variance analysis reveals significant relationships between ME ingredients and drug release rates:

2‐h drug release:

1. Oil and water percentages significantly affect the 2‐h drug release rate.
2. Decreasing the water percentage and increasing the oil percentage can boost the 2‐h release rate.
3. Formulation 2 shows the highest 2‐h release rate, while formulation 8 shows the lowest.

24‐h drug release:

1. Oil and water percentages significantly influence the 24‐h drug release rate of methimazole from MEs.
2. Higher oil percentage and lower water percentage in MEs increase the 24‐h drug release rate.
3. Formulation 2 (50% oil, 5% water, 45% S + C) demonstrates the highest 24‐h release rate.
4. Formulation 8 (50% oil, 10% water, 40% S + C) shows the lowest 24‐h release rate.

**FIGURE 4:**
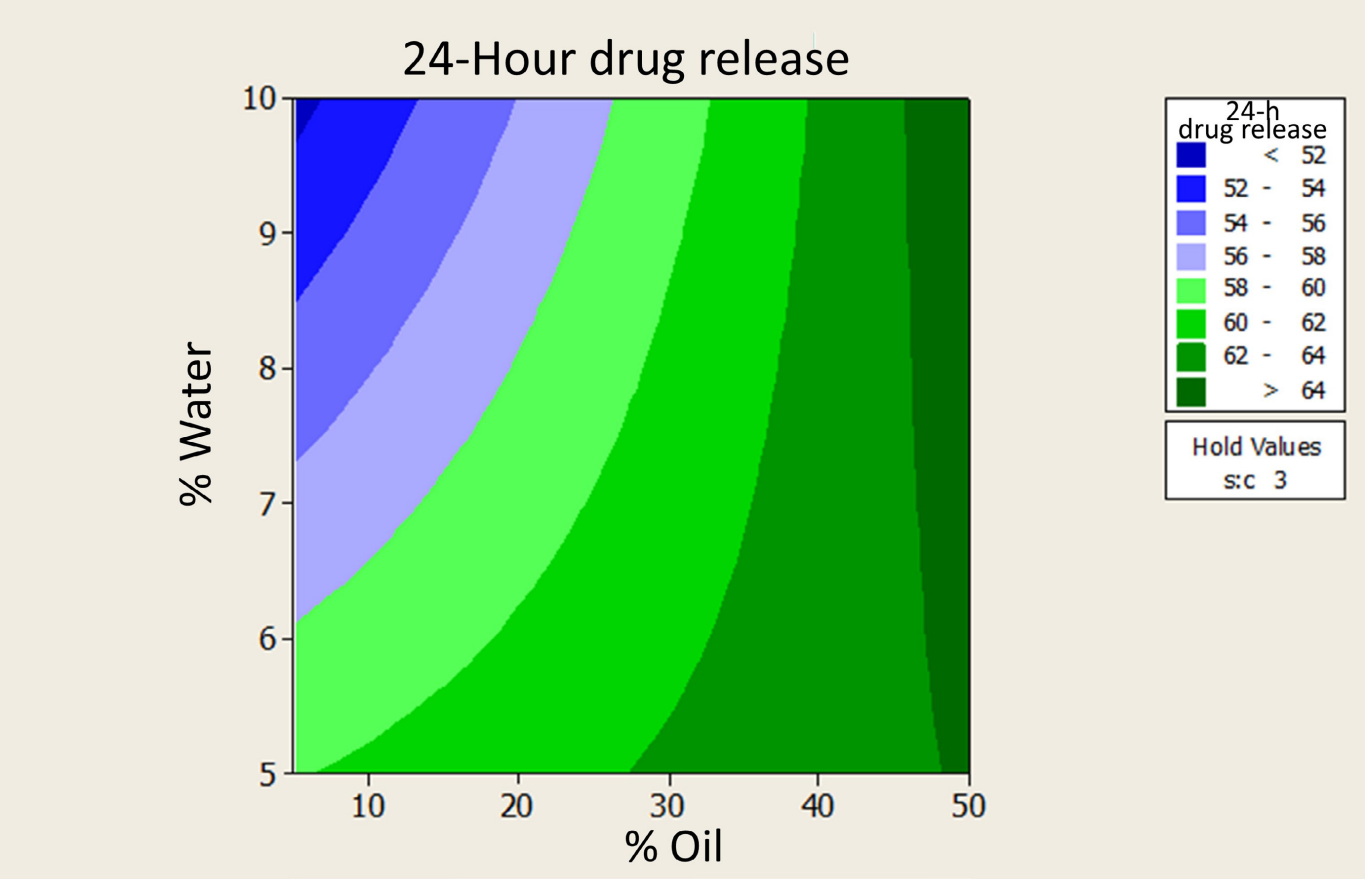
24‐h drug release percentage versus water and oil percentages in microemulsions.

Figure 4 illustrates the relationship between the 24‐h drug release percentage from MEs and the independent variables of water and oil percentages.

### 3.3 Permeability of methimazole MEs

Table 4 displays the permeability parameters of methimazole ME formulations compared to the drug saturation control using whole rat skin.

**TABLE 4:** Methimazole ME permeability through whole rat skin compared to drug control (*n* = 3, Mean ± SD).

Formulation	J_ss_ (mg/cm^2^.h)	D_app_ (cm^2^/h)	*p* (cm/h)	T_lag_ (h)	ER^a^_flux_	ER^a^_D_	ER^a^_p_
Control	0.0093 ± 0.0007	0.113 ± 0.009	0.031 ± 0.002	1.21 ± 0.11	‐	‐	‐
ME‐MTZ‐1	0.0190 ± 0.0002	0.136 ± 0.007	0.065 ± 0.001	0.99 ± 0.05	2.1 ± 0.2	1.2 ± 0.1	2.1 ± 0.2
ME‐MTZ‐2	0.0362 ± 0.0002	0.141 ± 0.004	0.120 ± 0.001	0.95 ± 0.03	3.9 ± 0.4	1.2 ± 0.1	3.9 ± 0.4
ME‐MTZ‐3	0.0222 ± 0.0002	0.089 ± 0.062	0.075 ± 0.001	2.01 ± 1.41	2.4 ± 0.3	0.8 ± 0.4	2.4 ± 0.3
ME‐MTZ‐4	0.0201 ± 0.017	0.218 ± 0.159	0.069 ± 0.059	0.84 ± 0.62	2.1 ± 1.7	2.0 ± 1.7	2.1 ± 1.7
ME‐MTZ‐5	0.0361 ± 0.0002	0.087 ± 0.001	0.119 ± 0.000	1.71 ± 0.03	3.9 ± 0.4	0.7 ± 0.1	3.9 ± 0.4
ME‐MTZ‐6	0.0232 ± 0.0003	0.146 ± 0.008	0.077 ± 0.001	0.92 ± 0.06	2.5 ± 0.3	1.3 ± 0.1	2.5 ± 0.3
ME‐MTZ‐7	0.0383 ± 0.0001	0.129 ± 0.002	0.128 ± 0.000	1.04 ± 0.02	4.2 ± 0.4	1.1 ± 0.1	4.2 ± 0.4
ME‐MTZ‐8	0.0341 ± 0.0001	0.142 ± 0.005	0.114 ± 0.003	0.95 ± 0.03	3.7 ± 0.3	1.3 ± 0.2	3.7 ± 0.3
ME‐Optimal	0.0343 ± 0.0001	0.785 ± 0.002	0.114 ± 0.012	0.17 ± 0.01	3.4 ± 0.1	6.4 ± 0.2	3.4 ± 0.1

^**a**^ *n* $\mathrm{ER}=\frac{\text{permeability parameter with}\;\mathrm{ME}-\mathrm{MTZ}\;\text{FORMULATION}\;}{\text{permeability parameter with Control}.}$

Our findings indicate that the microemulsion carrier increased the Jss (steady‐state flux) of all formulations:

- Formulation 7 (50% oil, 5% water, 45% s + c) showed the highest Jss.
- Formulation 1 (50% oil, 10% water, 40% s + c) had the lowest Jss.

The microemulsion carrier's effect on D (diffusion coefficient) varied among formulations:

- Formulation 4 exhibited the highest D value.
- Formulation 5 had the lowest D value.

### 3.4 Stability

We evaluated the stability of ME samples after 6 months of storage at 4°C, 25°C, and 37°C. Our observations revealed:

- No turbidity, precipitation, or phase separation in any samples.
- Drug content remained at 99.9%, indicating high chemical stability.
- Minimal changes in droplet size and pH, which did not significantly affect the results.

### 3.5 Optimal formulation

We used Minitab 16 statistical software to determine the optimal formulation through an optimization method. This process focused on two key factors:

- Droplet size.
- Percentage of drug release after 24 h.

We chose these factors as the most valid and effective data points. The method involved:

- Calculating the range between the lowest and highest data for each variable.
- Setting this range as the target.
- Identifying the best percentage for each phase that yielded our target values for particle size and 24‐h drug release.

The resulting combination was designated as the optimal formulation. Table 5 presents the optimization results:

**TABLE 5:** Optimization results and target value.

Parameter	Minimum	Maximum	Target
Droplet Size (nm)	7.06	49.20	28.13
24‐h release (%)	51.38	68.66	60.02

The optimal formulation of 3% methimazole was prepared using the values from the table. Its characteristics, including particle size and drug release percentage after 24 h (24% R), were measured. Table 6 presents these results.

**TABLE 6:** Optimal formulation values.

Water (%)	Oil (%)	The ratio of Surfactant to co‐surfactant (g)
50.2626	5.0944	2.5185

Variance analysis of the equation relating the formulation's particle size to independent variables is:

Droplet Size (nm) = −2.5 − 1.22 (%w) + 0.439 (%oil) + 12.2 (s/c).

This equation reveals a significant relationship between particle size, oil percentage, and the surfactant‐to‐cosurfactant ratio. As the oil percentage and surfactant to cosurfactant ratio increase, so does particle size.

The equation linking the 24‐h drug release percentage to independent variables is:

24‐h release (%) = 66.9–0.559 (%w) + 0.201 (%oil) − 2.69 (s/c).

This relationship indicates a significant connection between drug release percentage and oil and water percentages. The drug release rate rises with increasing oil percentage and decreasing water percentage.

We inserted the independent variable values from the optimization process into each equation to obtain the calculated value for each variable. These calculated values were then compared with the actual measured values.

Table 7 displays the calculated and real values, along with the regression *p*‐value for each variable.

**TABLE 7:** Characteristics of optimized methimazole ME formulation (Mean ± SD).

Parameter	The actual value based on the tests	The calculated value based on Equations	*p*‐value
Droplet Size (nm) (*n* = 3)	25.02 ± 0.01	25.24 ± 0.02	0.1
24‐h release (%) (*n* = 3)	55.71 ± 0.14	55.60 ± 0.24	0.1

The presented equations describing the relationship between droplet size characteristics and 24‐h drug release with independent variables show sufficient validity based on these results. The optimal formulation with suitable features has an optimization factor D equal to 1 for both droplet size variables and 24‐h release. This suggests that the optimal formulation is appropriate for conducting further tests in this study.

However, no significant relationship was found between the particle size and the 24‐h release of the optimal formula with calculated values.

We also examined the permeation rate of methimazole in the optimal pharmaceutical microemulsion through whole rat skin. Permeation parameters including J_ss_, p, T_lag_, and D_app_ were calculated. Table 5 (ME‐Optimal) presents these results. Additionally, we compared the ER_flux_, ER_D_, and ER_p_ of the optimal formulation to the water control (ME‐Optimal).

**FIGURE 5:**
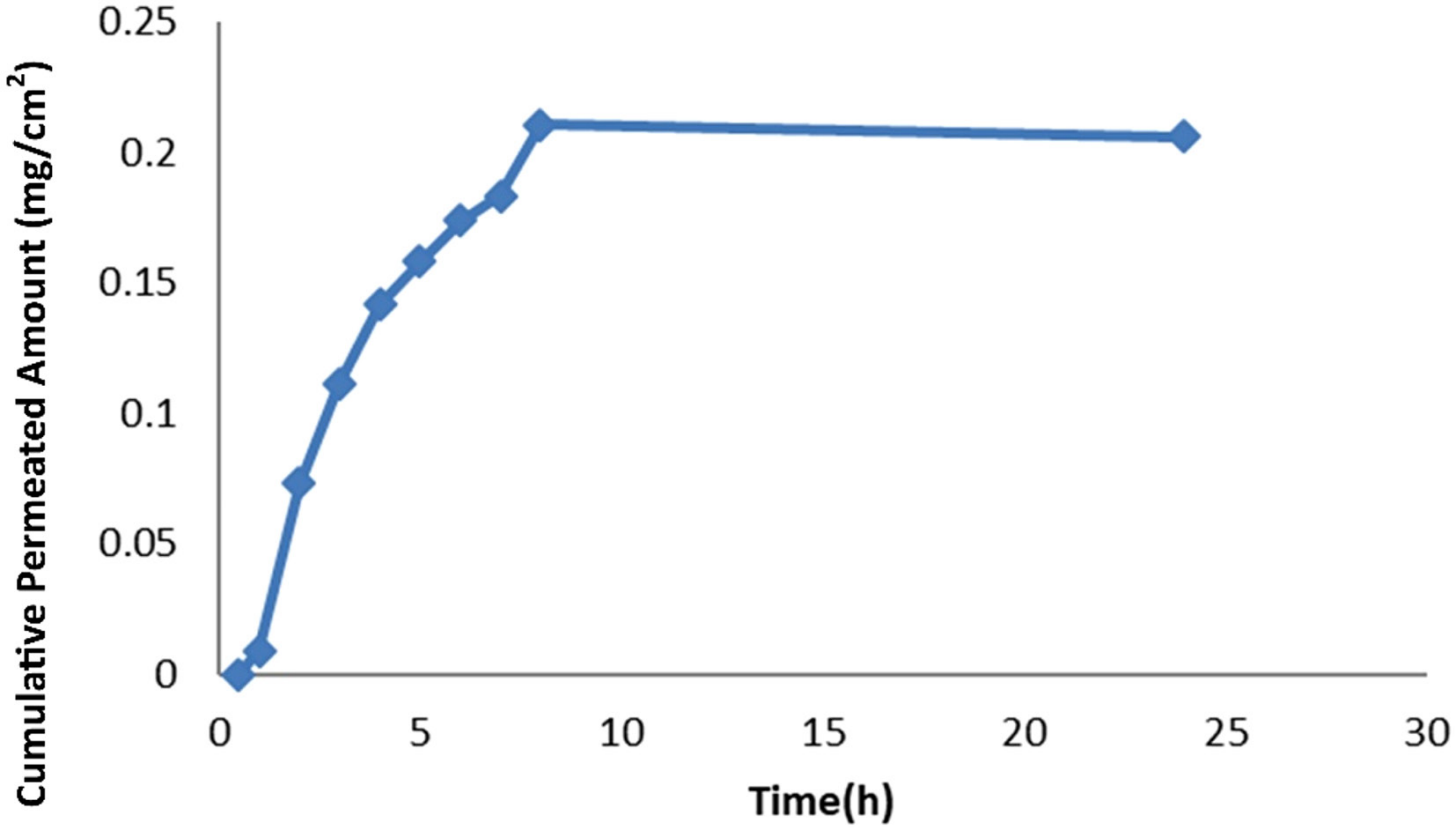
Cumulative methimazole permeation through rat abdominal skin from optimal microemulsion.

To determine permeability parameters, we plotted the cumulative amount of drug passed through the surface unit against time (Figure 5).

## 4 DISCUSSION

Methimazole, an anti‐hyperthyroid drug, acts as a depigmentation agent when applied topically by inhibiting peroxidase in skin melanocytes and preventing melanin production.^7^ Research by Kasraee et al. in 2008 showed that long‐term topical use of methimazole does not significantly affect serum thyroid hormone levels. Its minimal skin side effects and lack of cytotoxicity or mutagenicity make it a safe option for treating hyperpigmentation disorders.^22^ A subsequent study by Kasraee et al. found that daily application of 5% methimazole cream for 6 weeks reduced epidermal melanin in guinea pigs, suggesting its potential for improving hyperpigmented skin lesions.^23^

Our microemulsion evaluation revealed droplet sizes ranging from 9.33 to 47 nm, with dispersion indices below 0.5, indicating uniform droplet sizes across all formulations. Droplet size showed a significant positive correlation with oil percentage and s/c ratio.

Microemulsion viscosity ranged from 115 to 237 centipoise at 75 rpm shear speed. Viscosity increased significantly with higher oil percentage, lower water percentage, and reduced s/c ratio. Viscosity plays a crucial role in drug penetration through the skin and formulation stability. The formulations' rheological behavior at various shear rates appeared to follow Newtonian principles. Yuan et al. emphasized the importance of water, oil, and surfactant components in MEs.^24^

The pH of the prepared MEs ranged from 4.30 to 4.66, showing a significant inverse relationship with water percentage. Comparatively, Salimi et al. reported a pH of about 6.5 for celecoxib MEs using similar components.^25^ The same researchers found a pH of around 5.1 for azithromycin formulations.^26^ Mohammad Soleymani et al. reported a pH of about 5.4 for finasteride MEs with similar components.^15^ Kalantari et al. noted a pH of 4.8 for sour cherry kernel extract MEs.^27^

The drug release results after 2 and 24 h revealed significant relationships with water and oil percentages. Higher oil percentage and lower water percentage increased drug release rates at both time points. Formulation 2 showed the highest 24‐h drug release, while formulation 8 had the lowest. All formulations followed Higuchi model kinetics, indicating diffusion‐controlled release. This suggests that the rate‐limiting step for methimazole release from MEs is its release from oil droplets.^28^

MEs significantly increased ERflux, ERP, and ERD compared to the control (3% drug suspension) in rat skin permeation studies. However, Jss, P, incubation time, and Dapp showed no significant relationship with independent variables within the studied range. Increased drug permeability due to higher oil content may be attributed to oleic acid's absorption‐enhancing properties. Oleic acid disrupts stratum corneum lipid structure, increasing fluidity and penetration.^29^, ^30^, ^31^ It primarily enhances permeability through the non‐polar path by increasing both diffusion and absorption.^32^, ^33^ Oleic acid may also lower the lipid binding temperature, dissolving stratum corneum lipids.^34^

The microemulsion structure, particularly the increased oil phase and surfactant content, had a greater impact on flux and p values. This effect likely results from lipid matrix liquefaction or corneal tissue lipid structure disruption by oleic acid and the formulation's surfactant system.^15^

Previous studies have shown that unsaturated fatty acids, especially those with more double bonds and cis spatial arrangements, have stronger enhancing effects than saturated fatty acids. Oleic acid's unsaturated bond with a cis arrangement induces disorder in the intercellular bilayer structure, reducing the gel to liquid crystal transition temperature.^35^

Propylene glycol accelerates drug distribution in the stratum corneum and slightly disrupts cellular lipid structure. Using 10% propylene glycol with oleic acid enhances the permeation effect.^34^ It acts by solvating keratin in the stratum corneum and occupying hydrogen bonding sites.^36^

Non‐ionic surfactants alter drug distribution in the skin^37^ and increase penetration by dissolving stratum corneum lipids.^38^ Their protein‐binding ability in the stratum corneum enhances absorption properties and interferes with keratin cells. Span20, a non‐ionic surfactant, is a strong skin permeation enhancer.^15^

## 5 CONCLUSION

This study demonstrates that alterations in microemulsion content and composition significantly influence the physicochemical characteristics and permeability parameters of drugs in microemulsion formulations during rat skin penetration. The microemulsion carrier improved drug dispersibility across all formulations. Some carriers increased drug distribution up to tenfold compared to the saturated control. The optimal formulation presented in this article offers the most suitable proportions for potential market introduction.

## AUTHOR CONTRIBUTIONS

A.S. conceptualized and designed the evaluation and wrote the initial manuscript draft. S.M.S. contributed to the evaluation design, conducted part of the statistical analysis, and assisted in manuscript preparation. A.S. and S.M.S. jointly reassessed the data, refined the statistical analysis, and revised the manuscript. H.H. gathered and interpreted the clinical data, and contributed to manuscript revisions. All authors (A.S., S.M.S., H.H.) gave final approval for publication.

## CONFLICT OF INTEREST STATEMENT

The authors declare no conflict of interest.

## ETHICS STATEMENT

The ethics committee of Jundishapur University of Medical Sciences (IR.AJUMS.ABHC.REC.1397.087) on 13.03.2019 provided ethical approval for this study.

## DECLARATIONS

This paper has been approved by all the co‐authors and the responsible authorities at the institute where the research was carried out.

## Data Availability

The data that support the findings of this study are available from the corresponding author upon reasonable request.
